# Cost-utility analysis of maintenance therapy with gemcitabine or erlotinib *vs* observation with predefined second-line treatment after cisplatin–gemcitabine induction chemotherapy for advanced NSCLC: IFCT-GFPC 0502-Eco phase III study

**DOI:** 10.1186/1471-2407-14-953

**Published:** 2014-12-15

**Authors:** Isabelle Borget, Maurice Pérol, David Pérol, Armelle Lavolé, Laurent Greillier, Pascal Dô, Virginie Westeel, Jacky Crequit, Hervé Léna, Isabelle Monnet, Hervé Le Caer, Pierre Fournel, Lionel Falchero, Michel Poudenx, Fabien Vaylet, Sylvie Chabaud, Alain Vergnenegre, Gérard Zalcman, Christos Chouaïd, on behalf of the IFCT-GFPC investigators

**Affiliations:** Études et Recherche en Économie de la Santé, Service de Biostatistique et d’Epidémiologie, Institut Gustave Roussy, 114, rue Edouard-Vaillant, 94805 Villejuif, Cedex, France; Centre Léon Bérard, Lyon, France; Hospices Civils de Lyon, Lyon, France; Hôpital Tenon, AP-HP, Paris, France; Aix Marseille Université, Inserm, CRO2 UMR_S 911, Marseille, France; APHM, Hôpital Nord, Service d’Oncologie Multidisciplinaire et Innovations Thérapeutiques, Marseille, France; Centre Régional de Lutte contre le Cancer François Baclesse, Caen, France; Service de Pneumologie, CHU Besançon, Besançon, France; Service de Pneumologie, CH Creil, Creil, France; Service de Pneumologie, CHU de Rennes, Rennes, France; Service de Pneumologie, Centre Hospitalier Intercommunal de Créteil, Créteil, France; Service de Pneumologie de Draguignan, Draguignan, France; Département d’Oncologie Médicale, Institut de cancérologie Lucien Neuwith, Saint-Priest en Jarez, France; Service de Pneumologie, Villefranche sur Saône, France; Centre Régional de Lutte contre le Cancer Antoine Lacassagne, Nice, France; Service des Maladies Respiratoires, HIA Percy, Clamart, France; Service de Pneumologie CHU de Limoges, Limoges, France; Service de Pneumologie et Oncologie thoracique, Caen University Hospital, Caen, France; Hôpital St-Antoine, APHP and UPMC University Paris 06, Paris, France

**Keywords:** Cost-utility, Maintenance therapy, Erlotinib, Gemcitabine, Non-small-cell lung cancer

## Abstract

**Background:**

The IFCT-GFPC 0502 phase III study reported prolongation of progression-free survival with gemcitabine or erlotinib maintenance *vs.* observation after cisplatin–gemcitabine induction chemotherapy for advanced non-small-cell lung cancer (NSCLC). This analysis was undertaken to assess the incremental cost-effectiveness ratio (ICER) of these strategies for the global population and pre-specified subgroups.

**Methods:**

A cost-utility analysis evaluated the ICER of gemcitabine or erlotinib maintenance therapy *vs.* observation, from randomization until the end of follow-up. Direct medical costs (including drugs, hospitalization, follow-up examinations, second-line treatments and palliative care) were prospectively collected per patient during the trial, until death, from the primary health-insurance provider’s perspective. Utility data were extracted from literature. Sensitivity analyses were conducted.

**Results:**

The ICERs for gemcitabine or erlotinib maintenance therapy were respectively 76,625 and 184,733 euros per quality-adjusted life year (QALY). Gemcitabine continuation maintenance therapy had a favourable ICER in patients with PS = 0 (52,213 €/QALY), in responders to induction chemotherapy (64,296 €/QALY), regardless of histology (adenocarcinoma, 62,292 €/QALY, non adenocarcinoma, 83,291 €/QALY). Erlotinib maintenance showed a favourable ICER in patients with PS = 0 (94,908 €/QALY), in patients with adenocarcinoma (97,160 €/QALY) and in patient with objective response to induction (101,186 €/QALY), but it is not cost-effective in patients with PS =1, in patients with non-adenocarcinoma or with stable disease after induction chemotherapy.

**Conclusion:**

Gemcitabine- or erlotinib-maintenance therapy had ICERs that varied as a function of histology, PS and response to first-line chemotherapy.

## Background

The National Institutes of Health estimated that $89 billion were spent on cancer care in the US in 2007, and that the total economic burden reached $219.2 billion when indirect costs associated with lost productivity and premature death were taken into account. Recent trends suggest that the growth of cancer spending will accelerate, owing to costly new treatments [1] and the increasing number of cancer patients. Lung cancer is the second most common malignancy in the US and is the leading cause of cancer-related deaths [2]. Non-small-cell lung cancers (NSCLC) represent 80% of lung cancers and most patients already have advanced or metastatic disease at diagnosis. Chemotherapy with 4 to 6 cycles of a platinum-doublet is considered the standard of care for first-line treatment of eligible patients with advanced NSCLC. Most patients’ cancers progress after first-line therapy and second-line chemotherapy is recommended for those with performance-status (PS) 0 or 1 [3].

Maintenance therapy refers to therapy administered after the initial chemotherapy regimen and it is continued or maintained until progression. Multiple approaches are used, including continuation of a portion of the first-line therapy or “switching” to a non-cross–resistant chemotherapy [4]. The first studies conducted in this setting used chemotherapy agents [5–8]. Gemcitabine given to advanced NSCLC patients without progression after first-line treatment with a gemcitabine-cisplatin combination significantly improved progression-free survival (PFS), compared to the best palliative care, but the trial did not reach statistical significance for overall survival (OS) [5]. Early docetaxel [8] for non-progressive patients after a platinum doublet compared to delayed docetaxel achieved a 3-months prolongation of PFS with a not statistically significant increase in OS. In a similarly designed trial on pemetrexed, PFS and OS were significantly improved for the pemetrexed arm, with a survival advantage of 2.8 months [7]. More recently, the results of a phase III study [9] also showed a PFS benefit of continuing pemetrexed after 4 cycles of cisplatin–pemetrexed for advanced non-squamous NSCLC. Maintenance with targeted therapies also seems to be an attractive option, with bevacizumab [10, 11], cetuximab [12], erlotinib [13] and the erlotinib–bevacizumab combination *vs.* bevacizumab [14] yielding positive results. Recently, in a phase III trial, Pérol et al. [15] compared gemcitabine or erlotinib maintenance *vs.* observation for patients without disease progression after platinum-based first-line chemotherapy, with a predefined second-line therapy (pemetrexed), and found either gemcitabine or erlotinib to have a significantly improved PFS (primary endpoint), with a not significantly positive impact on OS, possibly by a lack of power.

Maintenance therapy for advanced NSCLC not progressing after a first cisplatin doublet became an option [3] but the cost-effectiveness of this strategy has not been well-established [16–20]. The published studies [16–19] had several limitations: maintenance-period costs collected retrospectively, second-line costs not collected [19, 20] or model-based study with no prospective recording of cost data [18, 19].

The purpose of this study was to conduct an economic analysis of the IFCT-GFPC 0502 trial [15], in which each patient’s consumed resources were collected prospectively from randomization (after induction treatment) to the end of the predefined pemetrexed second-line chemotherapy. The aim of this cost-utility analysis was to assess the incremental cost-effectiveness ratio (ICER) of either the gemcitabine or erlotinib strategy compared to observation, for all NSCLC patients who received maintenance therapy (not selected population), and in different patient subgroups, according to PS (0 or 1), histology (adenocarcinoma or non-adenocarcinoma) or response to first-line cisplatin-doublet chemotherapy (stable disease or responder).

## Methods

### Patients and treatment

**Figure 1:**
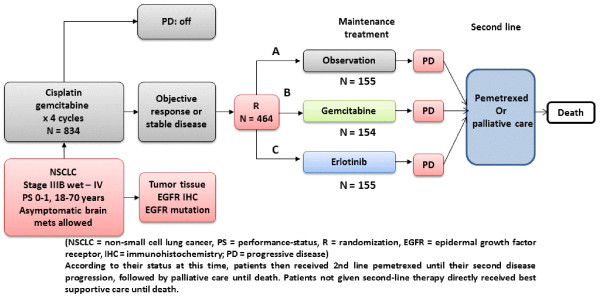
**Design of the phase III IFCT-GFPC 05-02 trial.** Legend: NSCLC, non-small-cell lung cancer; mets: metastasis; PD: progressive disease; PS: performance-status; EGFR IHC: epidermal growth-factor receptor detected by immunohistochemistry; CT: chemotherapy. R: randomization.

Patients’ data analyzed herein were obtained from the phase III IFCT-GFPC 05-02 trial [ClinicalTrials.gov identifier NCT00300586, 15]. All patients provided written informed consent for participation in the study and consent for tumor sample collection. The study was approved by the Ethics Committee of Lyon, France and was conducted in accordance with the Declaration of Helsinki and Good Clinical Practice guidelines. Briefly, the objective of that study was to investigate gemcitabine or erlotinib maintenance *vs.* observation for PS 0–1, advanced NSCLC patients, with no evidence of disease progression after 4 cycles of cisplatin–gemcitabine, and who received pemetrexed as the predefined second-line therapy (Figure 1). Of the 834 patients who received induction chemotherapy, 464 (55.6%) were randomized. Median PFS was significantly prolonged with gemcitabine (3.8 months) or erlotinib (2.9 months) compared to observation (1.9 months). The median PFS benefit seemed to be irrespective of histology for gemcitabine or erlotinib. Second-line pemetrexed was administered in 84%, 74% and 75% of the patients in the observation, gemcitabine and erlotinib arms, respectively. Median OS was improved with gemcitabine (12.1 months) or erlotinib (11.4 months), although not significantly, compared to observation (10.8 months).

### Economic evaluation

The primary objective of this economic analysis was to evaluate the ICERs of gemcitabine or erlotinib maintenance therapy *vs.* observation. Results are expressed as cost per quality-adjusted life years (QALY).

The natural course of NSCLC was divided into management periods: maintenance therapy, second-line pemetrexed, palliative care and death. During the maintenance period, patients were randomized to receive gemcitabine, erlotinib or observation until disease progression. According to their status at this time, patients then received second-line pemetrexed until their second disease progression, followed by palliative care until death. Patients not given second-line therapy directly received best supportive care until death. Each patient’s time spent in each period was calculated with the trial data.

Costs were estimated from the French health payer’s perspective. They were computed from randomization until the patient’s death or censoring date, and were limited to direct medical costs. Each patient’s resource consumptions were prospectively recorded in the case-report form until the end of second-line chemotherapy. That consumption included chemotherapy drugs (gemcitabine, erlotinib and pemetrexed), hospitalization for any reason and follow-up exams. Costs, expressed in euros (€) were based on the national health-insurance provider’s tariffs for diagnosis-related groups and national fees for ambulatory care, provided by the French Ministry of Health and the national health-insurance provider [21, 22]. Table 1 shows unit prices and tariff sources.

**Table 1:** **Unit cost and health-utility values used for the economic analysis**

Category	Tariff	Origin
**Costs**		
Gemcitabine	€0.24/mg	National reimbursement price [21]
Erlotinib 30-day supply (150 mg)	€2174.70	National reimbursement price [21]
Pemetrexed	€2.45/mg	National reimbursement price [21]
GCS-F	€185.80/inj	National reimbursement price [21]
Epoetin	€221.2/inj	National reimbursement price [21]
DRG for transfusion	€697.10	DRG 11th edition [22]
DRG for outpatient drug administration	€400.70	DRG 11th edition [22]
Palliative care (per month)	€2324 [€1627–€3021]	Chouaïd [23]
**Health-utility values**		
*Maintenance therapy*		
Observation	0.693 [0.46–0.88]	Nafees et al. [24]
IV chemotherapy (gemcitabine)	0.653 [0.26–0.78]	Nafees et al. [24]
Oral chemotherapy (erlotinib)	0.673 [0.27–0.80]	Nafees et al. [24]
*Second-line therapy*		
IV chemotherapy (pemetrexed)	0.653 [0.26–0.78]	Nafees et al. [24]
*Palliative care*	0.473 [0.19–0.56]	Nafees et al. [24]
*Death*	0	

*Abbreviation*: *DRG* diagnosis-related group.

Costs incurred during the palliative period were derived from a representative French nationwide sample of 428 patients, using chart review to assess the mean direct monthly cost of the first 18 months of NSCLC patient management [23]. Specifically, the costs included outpatient and inpatient services, care provision at skilled nursing facilities, outpatient and inpatient drugs and other medications, nursing care organization, home health visits and durable medical equipment. Assuming a yearly increment of 3%, one month of palliative care cost was 2,324 euros (2011 value). Transport and indirect costs (like sick leave) were not included in the analysis, because of insufficient data. No discount rate was applied, given the short life expectancy of these patients.

### Utility

Self-assessed health state (or utility) scores measure the individual’s preferences for specific outcomes, and are used to calculate QALY. Because they were not directly collected during the study, utility values were extracted from a community population-based study on advanced NSCLC in the UK [24] using the standard gamble interview to assess quality of life. Utility scores differed according to the period and the type of treatment received (intravenous (IV) chemotherapy, oral chemotherapy, none) (Table 1). Each patient’s QALY was then calculated, from randomization to death or censoring date, by multiplying the duration of each period by the corresponding period’s utility score.

### Statistical and sensitivity analyses

Median PFS and OS were estimated using the Kaplan–Meier method. The gemcitabine or erlotinib strategies were compared to observation with the log-rank test. All tests were two-sided.

An ICER was calculated as the ratio of the mean cost difference to the mean effect difference between each treatment strategy (gemcitabine or erlotinib) and observation.

The uncertainty and robustness of the model were evaluated in one-way sensitivity analyses, by varying chemotherapy costs and utility values over a range of likely values derived from confidence intervals (CI) or reasonable ranges, while keeping the other parameters constant.

A bootstrap method, consisting of a resampling procedure with replacement based on the generation of 10,000 replications of the ratio using traditional probabilistic sensitivity analysis (PSA), was used to obtain the non-parametric 95% CI for the ICER. Estimations obtained with the 10,000 bootstrap replications are presented in a radar screen format, with the *X*-axis showing the difference in effectiveness (QALY) and the *Y*-axis giving the cost difference between two strategies. Dots represent the 10,000 replications. Bootstrapping was used to determine the proportion of dots in each quadrant of the cost-effectiveness plane. The proportion of replications below the €100,000 per QALY threshold was also calculated. SAS software version 9.2 (SAS Inc, Cary, NC) was used for statistical analyses.

## Results

Between July 2006 and June 2009, among 834 patients enrolled at 73 centers in France and who received cisplatin–gemcitabine induction chemotherapy, 464 were subsequently randomized to observation (n = 155) or gemcitabine (n = 154) or erlotinib (n = 155) maintenance therapy. Baseline characteristics were well-balanced among treatment arms (Table 2). At the cut-off date for the primary endpoint (30 August 2010), median follow-up for all patients was 25.6 months.

**Table 2:** **Baseline characteristics of randomized patients**

Characteristic	Observation n = 155	Gemcitabine n = 154	Erlotinib n = 155
Median age (range, years)	59.8 (37–72)	57.9 (29–71)	56.4 (36–71)
Gender, n (%)			
Male	113 (72.9)	113 (73.4)	113 (72.9)
Female	42 (27.1)	41 (26.6)	42 (27.1)
ECOG PS at inclusion, n (%)			
0	78 (50.3)	73 (47.4)	81 (52.3)
1	77 (49.7)	81 (52.6)	74 (47.7)
ECOG PS at randomization, n (%)			
0	68 (44.2)	61 (40.1)	58 (37.9)
1	81 (52.6)	82 (53.9)	85 (55.6)
2	4 (2.6)	7 (4.6)	8 (5.2)
3	1 (0.6)	2 (1.3)	2 (1.3)
Unknown	1 (0.6)	2 (1.3)	2 (1.3)
Stage, n (%)			
IIIB	14 (9.2)	14 (9.3)	11 (7.4)
IV	139 (90.8)	137 (90.7)	137 (92.6)
Unknown	2 (1.3)	3 (1.9)	7 (4.5)
Brain metastases, n (%)	1 (0.6)	5 (3.2)	2 (1.3)
Smoking status, n (%)			
Current and former smokers	143 (92.3)	137 (89)	138 (89.0)
Never smoker*	12 (7.7)	17 (11.0)	17 (11)
Histology, n (%)			
Adenocarcinoma	103 (66.5)	101 (65.6)	97 (62.6)
Squamous cell carcinoma	30 (19.4)	34 (22.1)	27 (17.4)
Unknown	22 (14.2)	19 (12.3)	31 (20)
Response to induction chemotherapy, n (%)			
Objective response	82 (52.9)	81 (52.6)	82 (52.9)
Stable disease	73 (47.1)	73 (47.4)	73 (47.1)

*Abbreviations*: *ECOG* Eastern Cooperative Oncology Group, *PS* performance status.

*Defined as consumption of < 100 cigarettes throughout one’s entire lifetime.

**Figure 2:**
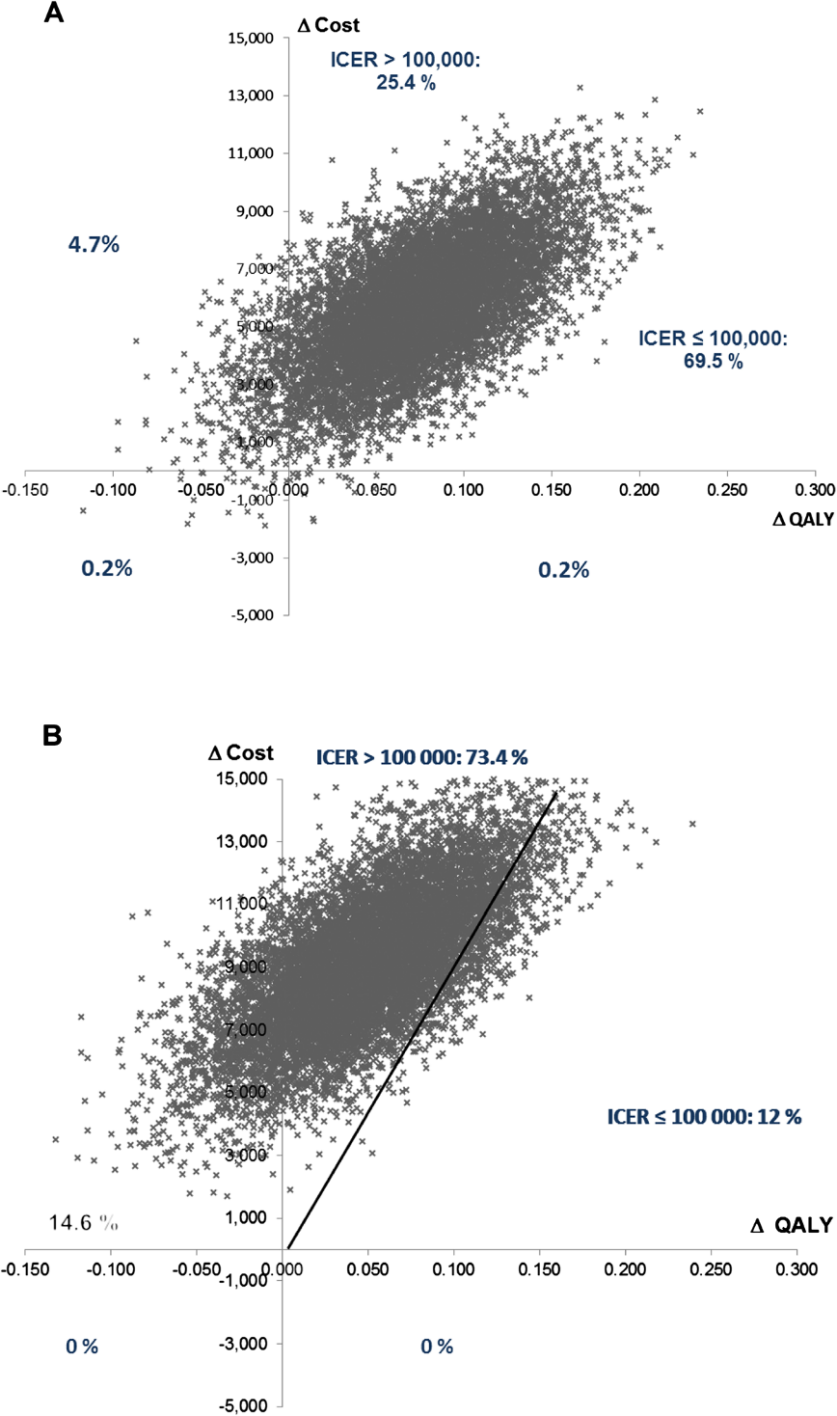
**Multivariate probabilistic sensitivity analysis (results of a 10 000-replication simulation). A**. Gemcitabine *v* observation, **B**. Erlotinib *v* observation.

The result of the economic analysis showed that the mean costs per patient of the maintenance period for gemcitabine, erlotinib or observation maintenance strategies were, respectively, €8,487, 10,551 and 702. With total costs respectively estimated at €28,397, 31,928 and 22,734 for the gemcitabine, erlotinib and observation strategies, maintenance therapy represented 30%, 33% and 3% of total management cost of each strategy (Table 3). Compared to observation, the analysis for the entire post-induction period showed that gemcitabine and erlotinib ICERs were, respectively, €76,625 [44,212 to 188,887] and €184,733 [94,559 to 1,186,736] per QALY. Estimations obtained with the 10,000 bootstrap replications showed that 94.9% of the dots were located in the quadrant in which the gemcitabine strategy was more effective and more expensive than observation, with an ICER ≤ €100,000 per QALY for 69.5% of the cases (Figure 2). For erlotinib maintenance, ICER was also more effective and more expensive than observation in 85.4% of the simulations but ≤ €100,000 per QALY for only 12% of cases.

**Table 3:** **Mean cost (in euros) and mean effectiveness (in QALY) per patient for each maintenance strategy, during the different management periods after induction chemotherapy**

	Observation (n = 155)		Gemcitabine (n = 154)		Erlotinib (n = 155)	
Parameter	Cost	QALY	Cost	QALY	Cost	QALY
Maintenance	€702	0.202	€8,487	0.256	€10,551	0.241
Second-line	€7,449	0.114	€6,412	0.103	€6,637	0.103
Palliative care	€14,582	0.229	€13,497	0.260	€14,741	0.251
Total	€22,734	0.545	€28,397	0.619	€31,928	0.595
Difference from observation	–	–	€5,663	0.074	€9,195	0.050
ICER	–	–	76,625 [44,212 – 188,887]		184,733 [94,559 – 1,186,736]	

Legend: ICER, incremental cost-effectiveness ratio; QALY, quality-adjusted life year.

Subgroup analyses (Table 4) showed that ICERs were favorable for the gemcitabine strategy, regardless of the histologic type, with PS = 0 or for patients with an objective response at the onset of maintenance therapy. The ICERs were also favorable for erlotinib in patients with PS = 0, who had an objective response to induction chemotherapy or with adenocarcinoma. Conversely, regardless of the treatment strategy, maintenance was not cost-effective for patients with PS = 1 or stable disease after induction. Additionally, receiving erlotinib was not cost-effective for patients with non-adenocarcInoma. The results of the one-way sensitivity analyses (Table 5) indicate that the main ICER drivers were the costs of maintenance treatments and health-utility values. In contrast, second-line medications and palliative care costs had only a weak impact on ICER.

**Table 4:** **Incremental cost-effectiveness ratio (ICER, expressed in euros per QALY), of the different patient subgroups**

Group	Gemcitabine (n = 154)	Erlotinib (n = 155)
All patients	76,625	184,733
Performance-status		
0	52,213	94,908
1	372,624	–*
Histology		
Adenocarcinoma	62,292	97,160
Squamous cell carcinoma or unknown	83,291	–*
Response to induction therapy		
Objective response	64,296	101,186
Stable disease	153,337	–*

Incremental cost-effectiveness ratios (ICER) are expressed in euros/quality adjusted-life years (QALY).

*The ICER was not calculated because the tested strategy does not appear to be more effective than observation.

**Table 5:** **Incremental cost-effectiveness ratio (expressed in euros per QALY) in the univariate analysis**

Parameter value	Gemcitabine (n = 154)	Erlotinib (n = 155)
Base case	76,625	184,733
Gemcitabine tariff		
–30%	47,132	–
Erlotinib tariff		
–30%	–	125,957
Pemetrexed tariff		
–30%	79,749	188,212
Palliative care cost		
€1,627 per month	81,021	183,772
€3,021 per month	72,213	185,680
Utility score under observation		
–10%	117,197	307 149
+10%	57,136	130,727
Utility score under IV chemotherapy		
–10%	€117,197	–
+10%	€57,136	–
Utility score under oral chemotherapy		
–10%	–	€366,092
+10%	–	€124,060

Incremental cost-effectiveness ratios (ICER) are expressed in euros/quality adjusted-life years (QALY). The sign “-” was used when the ICER was not affected by the univariate analysis.

Legend: IV: intravenous.

## Discussion and conclusion

This analysis of gemcitabine or erlotinib maintenance therapy *vs.* observation with a fixed second-line therapy for advanced NSCLC found a respective ICER of €76,625 and €184,733 per QALY but they varied widely depending on histology, PS and response to induction therapy. Gemcitabine for patients with PS = 0, objective response to induction, regardless of the histology, and erlotinib for patients with PS = 0, responses to induction or adenocarcinoma had the more favourable ICER.

Few economic studies on maintenance treatments for advanced NSCLC have been published [16–20]. Differences in methods, currency and economic outcomes, hindered direct comparisons with our results. In particular a majority of these studies reported a cost per life-year gained (LYG), which is not directly comparable to cost per QALY.

From the US primary health-insurance provider perspective, using the JMEN trial’s data [7], Medicare reimbursement rates and a retrospective claims database, Klein et al. [16] calculated the ICER of pemetrexed maintenance compared to observation and found, regardless of histologic subtype, an ICER of US$205,597 per life-year gained (LYG). This difference can mostly be explained by the cost of pemetrexed, which is much more expensive than gemcitabine. As in our study, their ICER varied according to histology, with US$122,371 per LYG for patients with non-squamous cell NSCLC. Using the same JMEN trial data [7] and based on the evidence submitted by the manufacturer, the National Institute for Health and Clinical Excellence (NICE) calculated an ICER of £47,000 per QALY for the non-squamous NSCLC population [25]. According to a model-based study from the Japanese health insurers vantage point [19], the ICER for pemetrexed maintenance was about US$109,024 per LYG and US$203,022 per QALY for all histologic types, again more favorable for non-squamous types (respectively, US$80,563 per LYG and US$150,115 per QALY).

Any studies have analyzed ICER according to the response to induction chemotherapy. In contrast to our findings, Vergnenègre et al. [26] conducted a model-based study using SATURN [13] data and found that the erlotinib cost per LYG versus best palliative care was €39,783, €46,931 and €27,885 in France, Germany, and Italy, respectively. This difference is mostly attributable to the methodology applied (retrospective analysis and modelization of second-line therapy costs). NICE analysis of the same strategy (erlotinib maintenance for patients with advanced NSCLC and stable disease after four cycles of platinum-induction therapy) calculated respective ICER of £44,812 and £68,120 per QALY for squamous and non-squamous NSCLC patients [27]. All these observations are compatible with our results for the total population, but their methodologies do not allow a precise analysis of the benefit of maintenance therapy according to important clinical characteristics, like PS and the response to induction therapy.

The latest guidelines recommend that quality of life be taken into account when considering treatment for NSCLC [28]. Taking account for the NSCLC burden in terms of health-related quality of life, little information is available on patients’ or society’s preferences with respect to disease states. We used data from Nafees et al. [24], who adapted existing health-state descriptions of metastatic breast cancer to evaluate the utilities of patients receiving second-line treatment for NSCLC. Each health state describes the symptom burden of a disease and its functional impact.

Our study had several strengths: a head-to-head comparative trial, prospective cost collection of data, patient cohort representative of those able to receive maintenance therapy in the routine clinical setting and a pre-specified second-line treatment administered in more than 75% of the patients in each arm. A subgroup analysis was performed as currently, systemic cytotoxic chemotherapy remains the first-line treatment for most patients with stage IV NSCLC, but preferred treatments are now defined by histology and based on the presence of specific molecular abnormalities [29]. It showed that ICERs of gemcitabine versus observation were more favorable in patients with PS = 0 or those who responded to induction compared to patients with PS = 1 or stabilization of disease after induction. For erlotinib arm, the tested strategy appeared to be not cost effective compared to observation for patients PS = 1, squamous histology or patients with stable disease as the better response after induction. However, it also had some limitations. First, costs were identified prospectively only during the active treatment periods, *i.e.* until the end of the second-line chemotherapy. Management costs after the end of active treatments were only derived from retrospective data of a 2004 national database. Some patients might have received and eventually benefit from a third-line chemotherapy, and we hypothesized that their expenditures for these chemotherapies would be identical in the 3 arms, and would not impact on the final results. Second, our analysis was limited to direct NSCLC-related medical costs: indirect costs, *e.g.* lost productivity and caregiver salaries, were not included. Third, the expression of utilities reflects the value from society’s perspective rather than that of the patients involved, since no prospective utility data were collected. Another point was that gemcitabine is now generic in most of the countries which likely means its costs were overestimated for gemcitabine strategy.

Finally at the time of the trial, pemetrexed was used for both squamous and non-squamous histology but only in second line setting. Now, pemetrexed is approved also for 1st line advanced NSCLC, but restricted to non-squamous histology. Therefore the trial population may be different than a potential gemcitabine or erlotinib maintenance population would be today.

Despite these limitations, the results of our analyses showed that gemcitabine or erlotinib maintenance therapy had acceptable ICER for certain subgroups, that they varied widely as a function of histology, PS and the response to first-line chemotherapy. These factors must be taken into consideration to better define the indications for NSCLC management.
